# Social Capital and Mental Health in Rural and Urban China: A Composite Hypothesis Approach

**DOI:** 10.3390/ijerph16040665

**Published:** 2019-02-25

**Authors:** Xiaoming Lin, Ruodan Lu, Liang Guo, Bing Liu

**Affiliations:** 1 Department of Statistics, Shandong University, Weihai 264209, China; xiaoming.lin@mail.sdu.edu.cn; 2 Department of Engineering, University of Cambridge, Cambridge CB2 1PZ, UK; rl508@cam.ac.uk; 3 School of Architecture, Building and Civil Engineering, Loughborough University, Loughborough LE11 3TU, UK; 4 School of Management, Shandong University, Jinan 250100, China

**Keywords:** China, human capital, multilevel linear regression, self-rated mental health, social capital

## Abstract

The objective of this study is to follow the composite theory approach to analyze the effect of social capital on self-rated mental health in rural and urban China. Our nationally representative sample includes 10,968 respondents from 130 county-level communities. Two-level random-coefficient linear regressions, which model individual and community variations in subjective mental health, were estimated by taking the hierarchical structure of the dataset into account. We found that a significant proportion of the total variations in self-rated mental health were explained at the community level. We also found an association between low contextual civic trust and poor self-rated mental health after adjusting for individual social capital and individual socioeconomic-demographic variables. The study also revealed that: (1) in rural areas a positive relationship between civic and political trust and mental health existed both at the individual and the community level, respectively; and (2) in urban areas, only political trust at the individual level contributed to better mental health. In addition, the individual and community level political participation exhibited a positive impact on mental health measures in both rural and urban China. The individual level civic participation was positively associated to the outcome variable. However, the community-level civic participation seemed to negatively impact mental health in urban area. Our findings emphasize the importance of both individual and community-level healthcare interventions in China. Finally, this study also found that human capital covariates remained important predictors of self-rated mental health status even after controlling social capital both at individual and community levels. This study suggested that the composite thesis could provide a more convincing narrative than other theories in explaining the effects of both human and social capital on health.

## 1. Introduction

As proposed by Sartorius [1] (p. 101), mental health is a ‘’the state of balance that individuals establish within themselves and between themselves and their social and physical environment’’. As per the report by Global Forum for Health Research and World Health Organization [2], mental and neurological disorders account for 13% of the global burden of disease. According to Zhang [3] and National Health and Family Planning Commission [4], 2.7% of the population in China was afflicted with severe mental illness in 1950s, a figure which rose to 5.4% in the 1970s, 11.1% in the 1980s, 13.47% in the 1990s, and further to 15% in the 2010s. Currently, over 30 million people have a diagnosis of severe mental illness (especially depression) in China [4]. Mental illness has been found to be the prime cause of the high suicide rate in China, which stands at approximately 10 per 100,000 in 2016 [5]. These statistics call for an urgent need to deepen our understanding on the determinants of Chinese mental health [6]. Research indicates that mental health is determined by socioeconomic, environmental, intersectoral, and civic security factors [7]. Mental health policy and practice remain pivotal to the discussion on social capital [8], because China’s surge in mental illness problems is probably due to its rapid urbanization, which is usually associated with a range of social health hazards and risks that can lead to the development of neuropsychiatric illness [4]. In fact, prior studies all over the world report that social capital is a crucial determinant of both physical and mental health issues at the individual and the aggregate level and their interactions [9,10,11,12,13,14,15,16,17,18,19]. Social capital can influence an individual’s health by opening information channels, promoting collective action, addressing detrimental cultural norms, and fostering the development of support systems serving as a source of self-esteem and mutual respect [14,20]. These mechanisms of social capital also work in the context of mental health [7,8].

However, most prior studies were focused on western developed economies and presented mixed findings [21]. Research in the transition economies alluded to a positive relationship between social capital and improved health [22]. As argued by Ichida et al. [23], the relationship between high social capital and good health indicators can vary owing to the cultural differences among countries, justifying the need for a systematic study in the different cultural contexts. Recently, relevant research has been conducted in East Asia (for example by Fujisawa et al. [24]; Suzuki et al. [25]; Miller et al. [26], Yip et al. [21], Sun et al. [27]; and Yamaoka [28]). Although these works have advanced our understanding of the social capital-general health nexus, little attention has been particularly devoted to compare how the influence of social capital on mental health behaviors. To our best knowledge, there is little research addressing how social capital influences mental health differently in Chinese urban and rural settings. Such study is important, as prior works indicate that there is a huge urban-rural health disparity in China and call for further studies [29,30,31,32].

The major objective of this study is to fill the above-mentioned gap. Specifically, we examined empirical relationships of four dimensions of social capital, i.e., civic participation, civic trust, political participation, political trust, on self-rated mental health. We followed the composite hypothesis, which argued that the mental health of an individual was influenced not only by his or her human capital (i.e., education, income and social status) but also by his or her social capital, as people are usually involved in a variety of analytically distinct but nonexclusive social networks [33,34,35]. We also followed the multi-level framework proposed in [36]. By community, we referred to counties (*xian* in Chinese) or districts (*qu* in Chinese), the third level of China’s administrative hierarchy. Our sample individuals were nested within communities and an individual’s mental health status was a function of a set of individual covariates and human capital (i.e., gender, age, education, marriage, unemployment, social-economic status, household annual income, household wealth and household size), individual-level social capital (i.e., civic trust, civic participation, political trust, political participation), and community-level social capital. The multilevel linear regression technique was chosen as the most appropriate design tool to explain the variation in mental health status of the population by both within-community and between-community differences. To our best knowledge, the sample size of our study is the largest of its kind at the individual and community levels. 

## 2. Materials and Methods 

### 2.1. Sample

We used the 2015 sociological survey dataset provided by China’s National Survey Research Center (the dataset can be found at http://cnsda.ruc.edu.cn/). Data were collected using a stratified clustered sampling design and were representative for the non-institutionalized adult (i.e., 18 and above) population in China. The primary survey unit (PSU) of this survey is at the county level or district level. Using China’s latest census as the sampling frame, the survey project included 969 of the most crowded PSUs across China. One hundred and thirty PSUs were randomly chosen and each chosen PSU was assigned a quota of households based on the population of that neighborhood as a proportion of the total population of all 969 PSUs. The researchers then selected households randomly using the residence records in the sampling frame. One adult person was randomly selected from each sampled household to serve as a respondent. Survey administrators visited each household after 18:00 h on weekdays or after 14:00 h during weekends and holidays to maximize the participation rate. The dataset consists of 10,968 face-to-face interviews (6470 respondents from rural and 4498 from urban areas, respectively). The sample includes 5834 female and 5134 male individuals from 130 PSUs (hereafter, “communities”) in 28 provinces. Most of the respondents were aged 41–60 (40%). Our samples were nested in two levels: the individual level and the community (i.e., county or district) level. The detailed distributions of sample are summarized in Table 1.

### 2.2. Outcome Variable: Self-rated Mental Health

Self-rated mental health status was based on five statements that are similar to the WHO-Five Well-Being Index (WHO-5): “I often feel calm and peaceful; I often feel active and vigorous; I often feel depressive and unhappy; I often feel exhausted and tired; and I often feel that I cannot stand my life”. For the first two statements, the responses were given a value from (1) “not at all” to (5) “very true”. As for the last three statements, the responses were given a value from (1) “very true” to (5) “not at all”. The Cronbach’s alpha was 0.86. The eigenvalue of one principal component of this scale is 3.29, which represents the significant amount of variance in each item that can be explained by the principal component. 

The items were evaluated by a confirmatory factor analysis (CFA) model. A weighted average mental health score was generated, which was resulting from the multiplication of each statement by its corresponding CFA standardized regression weight. The mental health score was standardized to have a mean of zero and a standard deviation of one. That is, if an individual is in good mental health status, then his or her mental health score is positive. Otherwise, it is close the zero or negative.

### 2.3. Independent Variables: Social Capital

Social capital is regarded as “connections among individuals in social networks and norms of reciprocity and trustworthiness that arise from them” [37] (p. 19). The literature has identified four dimensions of social capital:

Structural social capital refers to an individual’s social network and various forms of civic engagement while cognitive social capital means an individual’s subjective perception of level of trust and reciprocity, which can be regarded as the result of structural social capital [38,39,40,41,42]. Horizontal social capital (or bonding/bridging social capital) refers to the relations developed between individuals or groups at the same social-economic hierarchical level, while vertical social capital (or linking social capital) includes the vertical relations between individuals or groups at different levels of formal power or authority [39,43,44,45]. Finally, social capital is not just an individual’s resource [46,47,48] but also a contextual capital [39]. Community or neighborhood social capital is usually based on day-to-day interaction between individuals [49]. According to [50,51] and [52,53], an individual in a particular area is also exposed to the community-level context, which may have an effect on an individual’s health. Therefore, we included contextual social capital variables, which can be constructed by grouping individuals within the same county/district and by aggregating an individual’s answers.

In this study, we have adopted the four-dimension framework of [39] to measure both individual and community-level social capital in the Chinese context. 

The first dimension is civic participation (CP), a type of structural and horizontal social capital, here assessed by the frequency of an individual’s participation in nine different civic activities over the past 12 months (i.e., festivals, kinship networking, sports, entertainments, time with fellow workers, skill development, philanthropy, educational and religious activities). The responses were given a value from (1) “not at all” to (5) “very frequently”. The Cronbach’s alpha was 0.74. The eigenvalue of one principal component of this scale is 3.37, indicating a substantially large amount of variance in each item that can be explained by the principal component. The items were standardized to have a mean of zero and a standard deviation of one, and were then evaluated by a CFA model. A weighted average CP score was generated, which was resulting from the multiplication of each statement by its corresponding CFA standardized regression weight.

The second dimension is civic trust (CT), a type of cognitive and horizontal social capital, here measured by the degree of a respondent’s trust on people, based on 13 different types of relations (i.e., close neighbors, distant neighbors, people living in the same town/village with the same family name, people living in the same town/village with different family name, relatives, acquaintances, townsmen, members of gymnasium club, members of non-political associations, members of volunteer organizations, classmates, colleagues, and total strangers). For each statement, the responses were given a value from (1) “not true at all” to (5) “very true”. The Cronbach’s alpha was 0.81. The eigenvalue of one principal component of this scale is 4.03. The items were standardized to have a mean of zero and a standard deviation of one, and then evaluated by a confirmatory factor analysis (CFA) model.

The third level is political participation (PP), a type of structural and vertical social capital, here assessed using four binary items—whether the respondent was a member of the Chinese Communist Party (CCP) or any other political party, or a government official or military officer, or a member of People’s Congress (PC) or of People’s Political Consultative Conference (PPCC), or had participated in any political protests or petitions. The sum of these four items was used as the PP score, which was then standardized to have a mean of zero and a standard deviation of one. 

Fourthly, political trust (PT), a type of cognitive and vertical social capital, referred to the extent to which respondents trusted in China’s political institutions (i.e., CCP, PC, PPCC, court system, People’s Procuratorate, military, police, and local as well as central governments). PT was measured by nine statements in terms of a respondent’s satisfaction of political institution’s service performance on transparency and accountability, social inequality, healthcare disparity, social services for the elderly/poor households, education service, judiciary’s efficiency and fairness, crime crackdown, defense and policing, and environment protection. For each statement, the responses were given a value from (1) “not satisfied at all” to (5) “very satisfied”. The Cronbach’s alpha was 0.80. The eigenvalue of one principal component of this scale is 4.71. The items were standardized to have a mean of zero and a standard deviation of one, and then evaluated by a confirmatory factor analysis (CFA) model.

Confirmatory factor analysis was performed on the constructs of CP, CT, PT, and mental health using open source R statistics package (see www.r-project.org) to evaluate the factor structures. The results indicated a good fit of each construct yielding a root mean square error of approximation (RMSEA) smaller than 0.05 and goodness-of-fit index (GFI) as well as an adjusted GFI superior to 0.90. All items were significantly loaded on their latent variables. The internal consistency of the scales (i.e., Cronbach’s alpha) was greater than 0.70, indicating high reliability of our measures. 

Thereafter, we aggregated these four social capital subcomponents into one general social capital index (SCI). Finally, five contextual, district-level social capital variables (CP-Dist, CT-Dist, PP-Dist, PP-Dist, PT-Dist, and SCI-Dist) at the community level were measured on the basis of aggregated individual answers for each county/district.

### 2.4. Independent Variables: Human Capital Covariates

Following [54] proposition, we have included a series of socioeconomic-demographic variables to control the confounding effects.

Gender was represented by a dummy variable for female. Age was categorized into four categories (1 = 18–25; 2 = 26–40; 3 = 41–60, and 4 = 61 and above). Education was measured as the highest grade completed (0 = no formal education; 1 = primary school; 2 = junior high school; 3 = senior high school; 4 = college; 5 = postgraduate). Marriage was categorized into three groups (1 = never married; 2 = married; 3 = divorced or widows/widowers). Unemployment was a dummy variable for whether one had been unemployed for the last three months. Self-reported social-economic status was assessed by a five-point likelihood scale ranging from 1 (very low) to 5 (very high). The respondents were asked to compare their social-economic status with peers within the same community. 

Household income was measured based on the household annual expenditure (see [21]). Wealth index was constructed from a factor analysis of 15 terms related to household dwelling characteristics and ownership of consumer durables (such as, cars, TV sets, telephones, and so on. See [55] for more details. Finally, household size referred to the total number of family members that were living together.

### 2.5. Research Model

Previous study dichotomized output variable and used binominal logistic regression to estimate the effect of social capital. The benefit of the approach was to facilitate the interpretation of regression coefficient as the likelihood to report better or worse health condition. However, [39,56,57] argued that such a dichotomization of a continuous variable was often arbitrary, which could fail to provide complete information about the actual distribution of the dependent variable or even cause misleading results. Therefore, in this study, we have kept the outcome variable, i.e., self-rated mental health as continuous and used a series of two-level linear regressions to examine the effect of individual and community social capital on an individual’s mental health. Following the approaches of [58,59], and [21], we have specified the following basic model:

(1) $$MH_{ij}=\beta_{0j}+\beta_{1}\left(SC_{ij}-SC_{j}\right)+\beta_{2}SC_{j}+\beta_{3}X_{ij}+\mu_{0j}+\varepsilon_{ij}$$

where MH is the mental health status for individual i (level 1) in community j (level 2); SC is the set of social capital variables measured at both the individual and community levels; and X is a vector of socioeconomic-demographic variables. This model estimated the fixed β parameters, which represented the overall relationships between individual social capital variables, covariates and mental health. There were also two random parameters $\mu_{0j}$ (community level) and $\varepsilon_{ij}$ (individual level), which were assumed to follow a normal distribution and represent the differences from the corresponding means at both individual and community levels. The variance between-community and the variance between-individual within a given community were used to calculate the variance partition coefficient (VPC), which represented the deviation in mental health across individual and community levels [60]. That is to say, the proportion of the total residual variation that is due to differences between communities. As the community-level social capital variables were aggregated from those at the individual level, the value of individual social capital was group-centered in order to alleviate the multicollinearity problem (i.e., $SC_{ij}-SC_{j}$) [21,61,62].

**Figure 1:**
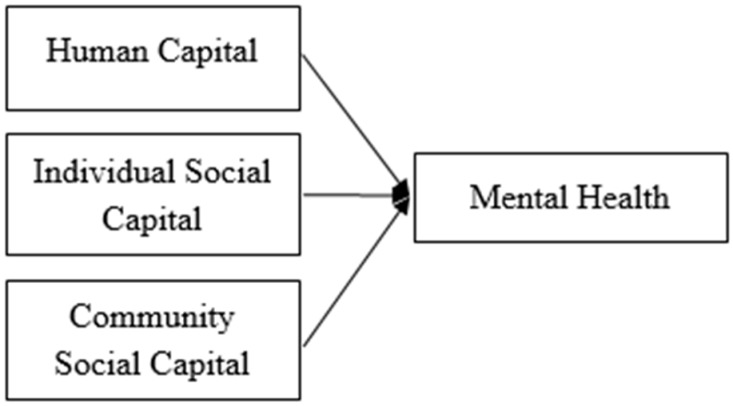
Research model.

Our research model is illustrated in Figure 1.

We have followed the nested modeling strategy of previous multilevel health studies (e.g., [16,21,42,56,63]) to account for the hierarchical structure of our dataset. We first estimated two variance components models (Model 0-Rural and 0-Urban) that include only random intercepts. Then we estimated Model 1-Rural and 1-Urban to include random intercepts and human capital variables. Finally, we estimated Models 2a–2e that include random intercepts, human capital variables, the five individual-level social capital variables (i.e., the aggregated SCI, CP, CT, PP, and PT) and the five community-level social capital variables (i.e., the aggregated SCI-Dist, CP-Dist, CT-Dist, PP-Dist, and PT-Dist), respectively.

## 3. Results

Table 1 summarizes the means (standard deviations) and correlations of all variables. We found that people in urban areas reported slightly better mental health status than their rural counterparts as indicated by mean values of 0.05(SD = 0.97) and –0.07(SD = 1.04), respectively. Urban individuals also outperform rural ones in terms of wealth index, household income, and education. However, urban individuals reported lower social-economic status, smaller household size and higher unemployment rate. In terms of social capital, it seems that urban people reported much higher civic participation (0.37 vs. −0.53) but much lower civic trust (−0.18 vs. 0.25), political participation (−0.21 vs. 0.3), and political trust (−0.07 vs. 0.10) than rural ones. In addition, it seems that mental health status is positively associated with all four types of social capitals and married, education, household income, household wealth and social-economic statute. However, mental health status is negatively associated with female and age.

Regression results of Model 0, Model 1, and Model 2a–2e are summarized in Table 2. Both the individual-level variances $\sigma_{\varepsilon }^{2}$ and the community-level variance $\sigma_{\mu }^{2}$ were all statistically significantly different to zero. The VPCs for the rural subsample ranged from 9.76% to 14.56%. And the VPCs for urban subsample were range from 9.76%. The VPCs indicate that substantial amount of vairance in mental health can be attributed to differences between communities. We then conducted the likelihood tests (see Table 3). We compared the loglikelihoods between Model 1 and 0. The differences (Δ-2ll) with 18 degrees of freedom were statistically significant under the Chi-square tests, suggesting that human capital variables as a whole are important determinants of mental health status in both rural and urban areas. Likewise, we compared Model 2a–2e to Model 1-Rural/1-Urban, respectively. The differences (Δ-2ll) with two degrees of freedom were statistically significant under the chi-squared tests in most models, except the one between Model 2b-Rural and Model 1-Rural. The results suggest that social capital as a whole and CT, PP, and PT are important determinants of mental health for both rural and urban individuals; while CP is an important determinant for urban individuals.

On closer inspection of Model 2a–2e, we noticed that the individual-level aggregated SCI was positively associated with mental health (0.01, *p* < 0.01) in both subsamples. However, the community-level aggregated SCI was positively associated with mental health only in the rural subsample (0.02, *p* < 0.05). In urban areas, the impacts of CP on mental health were significantly positive at the individual level (0.01, *p* < 0.01) but negative at the communitive level (−0.03, *p* < 0.01). The impacts of individual-level CT (0.1, *p* < 0.01) was statistically significant in rural area. The community-level CT had statistically significant impacts in both rural and urban areas (0.06 and 0.07, *p* < 0.01, respectively), which justified the contextual effects in [39].

What is more, the individual-level PP was positively associated (0.03, *p* < 0.01) with mental health in both rural and urban areas. Finally, the individual level PTs (0.02, *p* < 0.01; 0.02, *p* < 0.01) were positively associated with mental health in both rural and urban areas.

## 4. Discussion

This study follows the composite hypothesis to simultaneously test the effects of human and social capital on an individual’s self-rated mental health at both individual and community levels. The results of our multilevel linear regressions seem to support the composite hypothesis. We will discuss the impacts of social capital and human capital covariates on the outcome variable separately.

### 4.1. Effect of Social Capital

The study revealed that higher civic trust helped to improve the mental health at the individual (rural areas) and community level (both rural and urban areas). This finding further corroborated prior findings between positive health outcomes and trust [15,63,64,65,66,67,68], which suggested that higher civic trust may enhance social networking among individuals, thereby reducing the mental stress and then lead to better mental health status. 

Our study revealed that higher political trust helped to improve mental health at the individual and community level in rural areas. This finding was consistent with a prior study [69], which indicated that higher political trust could, one the one hand, led to an enhanced feeling of security [69] and created stronger affiliations with the legal system as identified through questions on government functioning; and on the other hand, higher political trust also promoted a better sense of belongingness and sense of responsibility [69]. 

We found that higher political participation also tended to improve mental health at the individual level in both rural and urban areas. That is probably that political participation may promote a sense of gratification from the social welfare activities and aroused deeper feelings of social and moral responsibility among individuals. It may be also instrumental in warding off socially deviant behavior among individuals, inculcating socially beneficial norms and promoting mental health. 

Finally, we revealed that higher civic participation helped to improve the mental health at the individual level in urban areas. That is probably that higher civic participation could be manifested through progressive coaching mechanism or knowledge dissemination process to fellow individuals, thereby leading to enhanced self-esteem among individuals. This could help in promoting social interactions, reducing stress, improving security networks, and thereby improving mental health. However, at the community level our study suggested that enhanced civic participation gave rise to reduced mental health in urban areas, ceteris paribus. An explanation to this could be drawn from the “network resources” approach [70]. As predicted in [71], subjective social status could be considered as a partly mediating factor in explaining the negative association between network resources and psychological stress. We found a positive correlation (0.36, *p* < 0.01, in Table 4) between subjective social-economic status and civic participation in our urban sample, suggesting that a higher level of community civic participation could create extra responsibility or overwhelming burden on an individual with relatively high social-economic status. This is especially true in China in where loss of “face” has been considered as a key deterrent in an individual’s access to social capital [20,72]. Individuals will stretch beyond their economic horizon to meet moral obligations and reciprocity norms in order to save “face” in their communities. Non-adherence to these norms would result in a disastrous solitary state leading to a lower mental health. However, as found in a prior study [20], over participation in social events could involve detrimental social norms like alcohol consumption, physical stress and inevitably reduced social family interactions, leading to increased mental stress and worsening mental health. 

### 4.2. Effect of Human Capital

Overall our findings were in line with those previously reported in developing countries that human capital factors were important predictors of self-rated mental health. The compositional effects remained statistically significant even after taking social capital variables into account. 

In particular, we found that an increase in age led to a lower self-reported mental health, which is of paramount importance when people reach their middle-age. That is probably because that responsibilities and senses of isolation or worthlessness may be bound to increase with age. We believe that this is especially true for the elderly in rural areas, when their adult children are away as migrant workers.

Female gender was also found to be related to poorer self-rated mental health in rural areas than in urban areas. That is probably because, on one hand, rural women may suffer from lower social status in their villages and on the other hand, it may be accepted that when husbands are away as migrant workers, the responsibility to take care of younger family members and elderly parents always falls on the women without anyone to share the load.

Secondary education was also found to improve self-rated mental health in both rural and urban areas. This is probably because education may enable individuals to better understand the benefits of a healthy lifestyle and healthcare utilization [56]. We noticed that when both individual and community-level social capital variables were present, college education exhibited a marginally significant, negative effect on self-rated mental health in rural areas. We believed that when compared to their urban counterparts, the college students from rural areas may have lesser chances to find decent jobs in China because their families possessed fewer social networks. This probably led to low economic returns to their college tuition fees and made them anxious and frustrated.

Our study also found that wealth mattered for mental health. Being rich or having relatively high social-economic status was strongly associated with superior self-rated mental health. We argued that this was probably because richer households or individuals may be able to access more medical care resources and experience less stress when compared to the poor, who had to work harder to earn their living.

An increase of household size in rural area was associated with a decrease in self-rated mental health status. Given family size in rural China is usually larger (mean = 4.47, in Table 4), this association may have had its origins in increased competition between siblings for parental attention, resources and extra burden to take care of elderly parents or even grandparents. However, an opposite effect was found in the urban sample. A possible interpretation of the positive family size–mental health relationship in urban China may be due to the strict one-child policy. With the family size being small in urban areas (mean = 3.47, in Table 4), children may enjoy greater parental attention in their early life and as a result, developed greater resilience to maladaptive responses and stressful events in adulthood. Likewise, a larger family size may increase the social contact of urban elderly, reducing their sense of loneliness, a general cause of many mental illnesses. 

In addition, our study indicated that, the married individuals portrayed better mental health when compared to unmarried individuals in rural areas. This finding was not supported in urban areas. We found that the mental health of the urban individual was found to deteriorate following a divorce or loss of spouse. 

We believe that there is a stark distinction in the levels of aspirations between the individuals hailing from rural and urban areas. In rural areas, the concept of close families, strong bonding, and well-knit relations may exist that foster easy solving of mutual discord and problems. However, in urban areas, individuals live with more autonomy in smaller nuclear families, which could be beneficial to the extent of lesser burdening an individual with monetary and social responsibilities. At the same time, smaller nuclear families could be detrimental since the individuals may face increased mental stress when handling problems since they lack the emotional support of spouse, leading to poor mental health and estranged family ties. Therefore, with the resulting non-successful matrimony or death of beloved partner, an individual in urban area may develop a sense of personal loneliness and dissatisfaction, lack of emotional stability, and a dearth of social interaction develops, resulting in reduced mental health. 

Finally, the employment status had no significant impact in rural areas. However, the effect of unemployment tended to be significantly negative in urban areas. We believe that the societal set up in rural and urban areas is starkly different. In rural areas, there are more closely-knit families characterized by large joint families. These families live together and most probably the family earnings are shared for the entire family’s sustenance. This involves collective earning and collective spending to supplement the entire family’s needs. This could be in contrast to the urban areas, where the concept of nuclear families with fewer members predominates. Financially independent individuals most probably prefer to be socially independent as well. Hence for individuals in urban areas, professional upheavals coupled with lack of emotional support may lead to mental dissatisfaction and reduced mental health.

## 5. Limitations and Conclusions

### 5.1. Limitations

Like most cross-sectional analyses, this study may be subject to common method bias as information regarding both social capital and mental-health is self-reported. Since both social capital and mental health can be expressed as an individual’s general well-being, there may be a recall bias [16,39]. In addition, the cross-sectional design usually leads to reversed causality problem—poor mental health may reduce one’s social capital. For example, participation in political activities requires inherent enthusiasm and good mental situation as a pre-requisite. A longitudinal study is desired to check the robustness of our findings. However, the aggregated measures of social capital and the use of mean-centered individual social capital can mitigate this problem as individual co-variance can be diluted [39]. On the other hand, the problem of reverse causality at the community level may not be an issue. Since the Chinese government restricts people’s freedom to choose which district to live in under the Household Registration System, the community-level measures of social capital may be exogenous and random.

### 5.2. Conclusions

Our results suggest that social capital is reflective of mental health both at the individual and community level. We have employed four cognitive-structural and horizontal-vertical constructs to comprehensively measure social capital in Chinese contexts and empirically test for observations across samples from rural and urban Chinese population segments. The results support the argument that in general, individual social capital holds a positive association with self-rated mental health in China. This study also finds that a significant proportion of the total variation in individual’s self-rated mental health status can be explained by differences at the community level. Those living in areas with low civic trust social capital have high excess risk of poor self-rated mental health. On the contrary, urban areas offer disagreement on the structural dimension measured through community-civic participation index, offering a negative association. An explanation for this has been based on the network resources approach. The likelihood ratio tests further support our conclusions that the impact of both individual and contextual social capital is not an artifact. These results offer further dimensions for future research. A more detailed and vigorous investigation may be called upon to study the impact of social capital variation, involving development of more suitable instruments to unearth the pattern of variations more systematically.

Finally, we suggest that policy mechanisms should be set in place to strengthen social networks and create economic upliftment of individuals. The programs should be aimed at inculcating social parity among the community members and at reducing the disparity in the access of medical benefits. Community health and social wellbeing for individuals should to be given attention with special emphasis to women, middle-aged, and elderly citizens. In rural areas, schemes should be set in place to encourage employment and business opportunities for youth through promotion of cottage industries. These schemes will be able to create employment avenues for the large number of college-educated students hailing from rural areas and hence avoid their rapid transition to larger cities. It will also be helpful in curtailing dissatisfaction among their elderly family members who are plagued with issues of staying alone as their children and even grand-children migrate to urban areas for better job prospects.

## Figures and Tables

**Table 1:** Descriptions of the sample.

Provinces/Regions	Urban	Rural	District/County (Community)	Male	Female	Avg. Age (SD)
Shanghai	502	0	8	236	266	54.89(17.93)
Yunnan	93	292	4	190	195	48.70(16.30)
Inner Mongolia	25	74	1	53	46	54.12(15.08)
Beijing	519	28	9	252	295	48.70(17.62)
Jilin	178	287	5	222	243	48.41(16.37)
Sichuan	275	291	6	268	298	55.12(16.04)
Tianjin	288	0	7	135	153	52.40(16.98)
Ningxia Hui	47	47	1	36	58	38.64(14.07)
Anhui	119	278	4	182	215	51.62(16.54)
Shandong	315	260	6	262	313	48.38(17.09)
Shanxi	189	91	3	137	143	44.84(16.63)
Guangdong	531	0	10	254	277	46.24(16.99)
Guangxi Zhuang	194	199	4	206	187	52.32(16.05)
Jiangsu	321	178	5	231	268	50.82(17.77)
Jiangxi	284	192	5	213	263	49.49(17.90)
Hebei	99	196	4	120	175	47.07(15.63)
Henan	216	366	6	259	323	50.97(17.36)
Zhejiang	341	121	5	202	260	52.22(18.09)
Hubei	350	250	6	274	326	51.91(15.41)
Hunan	240	235	5	222	253	52.80(17.24)
Gansu	50	145	2	88	107	49.47(15.52)
Fujian	194	100	3	147	147	50.51(16.80)
Guizhou	177	72	3	110	139	48.13(16.90)
Liaoning	345	50	4	189	206	49.49(17.27)
Chongqing	79	186	3	132	133	52.95(16.31)
Shaanxi	106	263	4	173	196	49.78(15.05)
Qinghai	75	26	1	51	50	49.67(14.30)
Heilongjiang	318	271	6	290	299	50.54(15.28)
Total	6470	4498	130	5134	5834	50.40(16.89)

**Table 2:** Results of regression models.

Model	0-Rural	0-Urban	1-Rural	1-Urban	2a-Rural	2a-Urban	2b-Rural	2b-Urban	2c-Rural	2c-Urban	2d-Rural	2d-Urban	2e-Rural	2e-Urban
Beta	−0.04(0.04)	0.10(0.03) **	−0.95(0.11) **	−1.13(0.09) **	−0.93(0.11) **	−1.07(0.09) **	−0.95(0.11) **	−1.10(0.09) **	−1.08(0.12) **	−1.23(0.09) **	−0.88(0.11) **	−1.06(0.09) **	−0.93(0.10) **	−1.13(0.09) **
Female			−0.05(0.03) *	0.08(0.02) **	−0.03(0.03)	0.08(0.02) **	−0.05(0.03) *	0.08(0.02) **	−0.04(0.03) *	0.08(0.02) **	−0.04(0.03)	0.08(0.02) **	−0.05(0.03) *	0.07(0.02) **
SocEco Statue.			0.15(0.01) **	0.16(0.01) **	0.15(0.01) **	0.15(0.01) **	0.15(0.01) **	0.16(0.01) **	0.15(0.01) **	0.16(0.01) **	0.15(0.01) **	0.16(0.01) **	0.15(0.01) **	0.16(0.01) **
Wealth			0.01(0.01) *	0.01(0.00) **	0.01(0.01) *	0.01(0.00) **	0.01(0.01) *	0.01(0.00) **	0.01(0.01) *	0.01(0.00) **	0.01(0.01) *	0.01(0.00) **	0.01(0.01) *	0.01(0.00) **
Relatively low			0.10(0.03) **	0.07(0.04) *	0.09(0.03) **	0.06(0.04) *	0.10(0.03) **	0.07(0.04) *	0.10(0.03) **	0.06(0.04) *	0.09(0.03) **	0.06(0.04)	0.09(0.03) **	0.07(0.04) *
Modest oncome			0.17(0.04) **	0.09(0.04) **	0.19(0.04) **	0.09(0.04) *	0.16(0.04) **	0.09(0.04) **	0.16(0.04) **	0.09(0.04) *	0.17(0.04) **	0.08(0.04) *	0.16(0.04) **	0.09(0.04) **
High oncome			0.17(0.07) **	0.09(0.04) *	0.19(0.07) **	0.09(0.04) *	0.16(0.07) **	0.09(0.04) *	0.17(0.07) **	0.09(0.04) *	0.16(0.07) **	0.08(0.04) *	0.17(0.07) **	0.10(0.04) *
Household size			−0.01(0.01) *	0.02(0.01) **	−0.01(0.01) *	0.02(0.01) **	−0.01(0.01) *	0.02(0.01) **	−0.01(0.01) *	0.02(0.01) **	−0.01(0.01) *	0.02(0.01) **	−0.01(0.01) *	0.02(0.01) **
Age 26–40			−0.29(0.06) **	−0.09(0.05) *	−0.30(0.06) **	−0.08(0.05) *	−0.29(0.06) **	−0.08(0.05)	−0.29(0.06) **	−0.09(0.05) *	−0.31(0.06) **	−0.10(0.05) *	−0.29(0.06) **	−0.09(0.05) *
Age 41–60			−0.38(0.07) **	−0.18(0.06) **	−0.40(0.07) **	−0.17(0.06) **	−0.38(0.07) **	−0.16(0.06) **	−0.39(0.07) **	−0.18(0.06) **	−0.41(0.07) **	−0.21(0.06) **	−0.39(0.07) **	−.18(.06) **
Age 61+			−0.28(0.08) **	−0.10(0.06) *	−0.30(0.07) **	−0.10(0.06) *	−0.28(0.08) **	−0.08(0.06)	−0.29(0.07) **	−0.10(0.06) *	−0.30(0.08) **	−0.14(0.06) *	−0.29(0.07) **	−0.10(0.06) *
Primary school			0.04(0.04)	0.00(0.06)	0.03(0.04)	−0.01(0.06)	0.04(0.04)	−0.00(0.06)	0.04(0.04)	0.01(0.06)	0.03(0.04)	−0.01(0.06)	0.04(0.04)	0.00(0.06)
Junior high			0.08(0.05) *	0.10(0.06) *	0.06(0.05)	0.08(0.06) *	0.07(0.05) *	0.09(0.06) *	0.08(0.05) *	0.11(0.06) *	0.06(0.05)	0.07(0.06)	0.08(0.05)	0.10(0.06) *
Senior high			0.05(0.06)	0.12(0.06) *	0.02(0.06)	0.10(0.06) *	0.03(0.06)	0.11(0.06) *	0.04(0.06)	0.13(0.06) *	0.02(0.06)	0.093(0.057) *	0.05(0.06)	0.12(0.06) *
College			−0.18(0.17)	0.12(0.06) *	−0.24(0.17)	0.08(0.06)	−0.21(0.18)	0.09(0.06) *	−0.20(0.17)	0.12(0.06) *	−0.20(0.17)	0.08(0.06)	−0.18(0.17)	0.12(0.06) *
Postgraduate			0.00(0.00)	0.01(0.20)	0.00(0.00)	−0.03(0.20)	0.00(0.00)	−0.03(0.20)	0.00(0.00)	0.01(0.20)	0.00(0.00)	−0.03(0.20)	0.00(0.00)	0.03(0.20)
Married			0.16(0.07) *	0.01(0.05)	0.16(0.07) *	0.00(0.05)	0.17(0.08) *	0.01(0.05)	0.15(0.07) *	0.00(0.05)	0.14(0.07) *	−0.01(0.05)	0.16(0.07) *	0.00(0.05)
Divorced/Widow			−0.06(0.10)	−0.14(0.07) *	−0.06(0.10)	−0.14(0.07) *	−0.05(0.10)	−0.14(0.07) *	−0.07(0.10)	−0.14(0.07) *	−0.07(0.10)	−0.15(0.07) *	−0.06(0.10)	−0.14(0.07) *
Unemployed			−0.15(0.14)	−0.10(0.03) **	−0.14(0.14)	−0.09(0.03) **	−0.15(0.14)	−0.09(0.03) **	−0.15(0.14)	−0.09(0.03) **	−0.14(0.14)	−0.09(0.03) **	−0.15(0.14)	−0.10(0.03) **
SCI-Indi.					0.01(0.00) **	0.01(0.00) **								
CP-Indi							0.01(0.01)	0.01(0.00) **						
CT-Indi									0.014(0.01) **	0.00(0.00)				
PP-Indi											0.03(0.01) **	0.03(0.01) **		
PT-Indi													0.02(0.01) **	0.01(0.00) **
SCI-Dist					0.02(0.01) *	−0.01(0.01)								
CP-Dist							0.01(0.02)	−0.03(0.01) **						
CT-Dist									0.06(0.03) **	0.07(0.02) **				
PP-Dist											0.00(0.03)	−0.01(0.02)		
PT-Dist													0.02(0.02)	−0.02(0.01)
$\sigma_{\mu }^{2}$	0.15(0.03) **	0.12(0.02) **	0.10(0.02) **	0.08(0.01) **	0.09(0.02) **	0.08(0.01) **	0.10(0.02) **	0.08(0.01) **	0.09(0.02) **	0.07(0.01) **	0.10(0.02) **	0.08(0.01) **	0.09(0.02) **	0.08(0.01) **
$\sigma_{\varepsilon }^{2}$	0.88(0.02) **	0.88(0.02) **	0.74(0.02) **	0.74(0.01) **	0.74(0.02) **	0.74(0.01) **	0.74(0.02) **	0.74(0.02) **	0.74(0.02) **	0.74(0.01) **	0.74(0.02) **	0.74(0.01) **	0.74(0.02) **	0.74(0.01) **
−2ll	11,758.18	16,806.03	11,014.44	15,677.74	10,978.14	15,651.57	11,013.29	15,662.8	10,999.03	15,658.99	11,000.53	15,650.52	10,997.09	15,669.1
VPC	14.56%	12.00%	11.90%	9.76%	10.84%	9.76%	−11015.59	9.76%	10.84%	8.64%	11.90%	9.76%	10.84%	9.76%

** *p* <0.01; * *p* <0.05. Standard errors in parentheses. $\sigma_{\varepsilon }^{2}$: individual-level variances; $\sigma_{\mu }^{2}$: community-level variance. SCI: social capital index; CP: civic participation; PP: political participation; PT: political trust; VPC: variance partition coefficient. Indi: Individual-level. Dist: District-level.

**Table 3:** The likelihood chi-squared tests.

Model Comparison	Δ-2ll	df	*p*-Value
1-Rural vs. 0-Rural	−743.74	18	<0.01
1-Urban vs. 0-Urban	−1128.29	18	<0.01
2a-Rural vs. 1-Rural	−36.3	2	<0.01
2a-Urban vs. 1-Urban	−26.17	2	<0.01
2b-Rural vs. 1-Rural	−1.15	2	>0.10
2b-Urban vs. 1-Urban	−14.94	2	<0.01
2c-Rural vs. 1-Rural	−15.41	2	<0.01
2c-Urban vs. 1-Urban	−18.75	2	<0.01
2d-Rural vs. 1-Rural	−13.91	2	<0.01
2d-Urban vs. 1-Urban	−27.22	2	<0.01
2e-Rural vs. 1-Rural	−17.35	2	<0.01
2e-Urban vs. 1-Urban	−8.64	2	<0.05

Δ-2ll = The –2ll value of the first model minus the –2ll value of the second model.

**Table 4:** Descriptive statistics and correlation matrix.

Rural\Urban	Rural (mean, SD)	Urban (mean, SD)	1	2	3	4	5	6	7	8	9	10	11	12	13	14
1. Female	0.51 (0.5)	0.53 (0.5)		−0.01	−0.01	−0.03 **	−0.02	−0.02	−0.15 **	0.02	0.07 **	−0.07 **	−0.08 **	−0.02	−0.08 **	0.00
2. Soc-Economic Status	7.18 (2.15)	6.64 (2.12)	0.01		0.41 **	0.25 **	0.02	−0.13 **	0.26 **	−0.00	−0.17 **	0.36 **	0.28 **	0.11 **	0.09 **	0.08 **
3. Wealth Index	−2.75 (3.21)	1.93 (4.87)	−0.02	0.41 **		0.35 **	0.02	−0.11 **	0.37 **	0.03 *	−0.10 **	0.24 **	0.36 **	−0.01	0.10 **	0.01
4. HouseholdIncome	9211.39 (10,639.28)	25,842.81 (44,648.74)	−0.05 **	0.35 **	0.51 **		0.01	−0.08 **	0.18 **	−0.02	−0.08 **	0.11 **	0.21 **	−0.01	0.05 **	0.01
5. HouseholdSize	4.47 (1.97)	3.47 (1.65)	−0.01	0.08 **	0.13 **	0.10 **		0.00	−0.10 **	0.08 **	0.03 *	0.02	−0.04 **	−0.02	−0.02	0.02
6. Age	44.73 (13.81)	44.68 (15.45)	−0.11 **	−0.07 **	−0.12 **	−0.10 **	0.03		−0.41 **	0.24 **	−0.05 **	−0.34 **	−0.28 **	0.02	0.18 **	−0.00
7. Education	1.33 (0.91)	2.4 (1.11)	−0.22 **	0.22 **	0.30 **	0.23 **	−0.04 **	−0.31 **		−0.07 **	−0.10 **	0.27 **	0.42 **	0.04 **	0.08 **	−0.04 **
8. Married	0.9 (0.31)	0.8 (0.4)	0.10 **	0.10 **	0.06 **	0.04 *	0.07 **	0.05 **	−0.00		0.07 **	−0.02	−0.13 **	0.03 *	0.12 **	−0.02
9. Unemployed	0.01 (0.1)	0.14 (0.34)	−0.02	−0.00	0.04 *	0.03	0.00	−0.03 *	0.04 *	−0.04 *		−0.10 **	−0.14 **	−0.06 **	−0.08 **	−0.03 *
10. MentalHealth	−0.07 (1.04)	0.05 (0.97)	−0.06 **	0.34 **	0.25 **	0.20 **	−0.00	−0.28 **	0.25 **	0.05 **	0.01		0.18 **	0.11 **	0.07 **	0.06 **
11. CP	−0.53 (0.5)	0.37 (1.09)	−0.10 **	0.14 **	0.23 **	0.22 **	0.01	−0.17 **	0.32 **	−0.10 **	0.04 **	0.11 **		0.07 **	0.15 **	0.03
12. CT	0.25 (0.98)	−0.18 (0.97)	−0.06 **	0.05 **	0.04 *	0.04 *	−0.02	0.05 **	0.05 **	0.03	−0.01	0.08 **	0.04 **		0.07 **	0.07 **
13. PP	0.3 (1)	−0.21 (0.95)	−0.16 **	0.13 **	0.07 **	0.08 **	−0.01	0.07 **	0.13 **	0.08 **	−0.02	0.11 **	0.12 **	0.10 **		0.08 **
14. PT	0.1 (0.99)	−0.07 (1)	−0.03	0.05 **	−0.06 **	−0.01	0.01	0.03 *	−0.02	−0.02	−0.01	0.04 *	0.05 **	0.06 **	0.12 **	

** *p* <0.01; * *p* <0.05. The upper triangular part of the matrix contains the correlation coefficients of the urban subsample. The lower triangular part of the matrix contains the correlation coefficients of the rural subsample.
